# Access to MRI for patients with cardiac pacemakers and implantable cardioverter defibrillators

**DOI:** 10.1136/openhrt-2021-001598

**Published:** 2021-05-14

**Authors:** Christopher Pieri, Anish Bhuva, Russell Moralee, Aderonke Abiodun, Deepa Gopalan, Giles H Roditi, James C Moon, Charlotte Manisty

**Affiliations:** 1 Institute of Health Sciences, Queen Mary University of London Barts and The London School of Medicine and Dentistry, London, UK; 2 Department of Cardiology, Barts Health NHS Trust, London, UK; 3 Department of Radiology, Imperial College London, London, UK; 4 Department of Radiology, University of Glasgow, Glasgow, UK; 5 Department of Cardiovascular Imaging, Barts Heart Centre, London, Greater London, UK

**Keywords:** magnetic resonance imaging, pacemaker, artificial, defibrillators, implantable, diagnostic imaging

## Abstract

**Objective:**

To determine provision of MRI for patients with cardiac implantable electronic devices (CIEDs; pacemakers and defibrillators) in England, to understand regional variation and assess the impact of guideline changes.

**Methods:**

Retrospective data related to MRI scans performed in patients with CIED over the preceding 12 months was collected using a structured survey tool distributed to every National Health Service Trust MRI unit in England. Data were compared with similar data from 2014/2015 and with demand (estimated from local CIED implantation rates and regional population data by sustainability and transformation partnerships (STPs)).

**Results:**

Responses were received from 212 of 223 (95%) hospitals in England. 112 (53%) MRI units’ scan patients with MR-conditional CIEDs (10% also scan non-MR conditional devices), compared with 46% of sites in 2014/2015. Total annual scan volume increased over fourfold between 2014 and 2019 (1090 to 4896 scans). There was widespread geographical variation, with five STPs (total population >3·5 million representing approximately 25 000 patients with CIED) with no local provision. There was no correlation between local demand (CIED implantation rates) and MRI provision (scan volume). Complication rates were extremely low with three events nationally in 12 months (0·06% CIED–MRI scans).

**Conclusions:**

Provision of MRI for patients with CIEDs in England increased over fourfold in 4 years, but an estimated 10-fold care gap remains. Almost half of hospitals and 1 in 10 STPs have no service, with no relationship between local supply and demand. Availability of MRI for patients with non-MR conditional devices, although demonstrably safe, remains limited.

**Key questions**

**What is already known about this subject?**

- Patients with cardiac implantable electronic devices (CIEDs) have a 75% lifetime chance of requiring an MRI scan; however, many report difficulties accessing scans despite having MRI-conditional devices.
- Previous data found that less than half of National Health Service hospitals in England offer MRI scans to cardiac device patients, while data from the USA suggest that patients with CIED are up to 50 times less likely to be referred for clinically indicated MRI scans.
- MRI scans in patients with CIEDs (including non-MR conditional) are low risk, provided appropriate protocols are followed, with recent guidelines promoting MRI where clinically indicated.

**What does this study add?**

- Provision of MRI scans to patients with CIED has improved with a fourfold increase in scan volume over 4 years; however, there are still clear inequities with significant geographic variation that is unrelated to demand and an estimated 10-fold under provision.
- This study reinforces the low risk of MRI in patients with CIEDs irrespective of type, provided appropriate protocols are followed.

**How might this impact on clinical practice?**

- MRI is integral to care pathways across multiple specialities (neurology, orthopaedics, oncology, urology).
- These data show that despite recent improvements, patients with CIED still face significant inequity of access to MRI with likely impact on clinical outcomes.
- Scanning patients with CIEDs is low risk but barriers persist due to the challenges of cross-disciplinary working between radiology and cardiology. These data should allay fears regarding risk of MRI in patients with CIED and highlight the gaps in provision, which require prompt intervention to address inequities in care that patients with CIED currently face.

## Introduction

Current clinical pathways across multiple domains (neurology, orthopaedics and oncology) include MRI for diagnosis and treatment planning. The presence of a cardiac implantable electronic devices (CIED—pacemaker or implantable cardiac defibrillator (ICD)) was previously considered an absolute contraindication to MRI due to a perceived risk,^1^ resulting in delays and potentially impacting clinical outcomes. This is a growing problem—there are an estimated 400 000 patients with CIEDs in England with 50 000 new CIED implants annually and an expanding list of clinical indications for MRI across healthcare.^2–5^

Recent developments have, however, made scanning possible in almost all cases. ‘MR-conditional’ devices are now standard of care for new CIEDs in the UK, and recent studies have demonstrated MRI scanning for patients with legacy (non-MR conditional) CIEDs to be low risk, provided strict protocols are adhered to.^6 7^ However, patients with CIED report difficulties accessing MRI services worldwide.^7 8^ An initial survey conducted in 2015 found that although awareness of MR-conditional devices was high, fewer than half of hospitals offered a service with an estimated 50-fold national under provision.^9^ Reported barriers to service provision included safety concerns and practical issues related to inadequate cardiology support.

Since 2016, the landscape has changed with landmark clinical safety studies for non-MR conditional (‘legacy’) device scanning,^6 10 11^ international guidelines for best practice,^8 12–15^ funding approval by Medicare and Medicaid for MRI in almost all patients with CIED (including non-MR conditional devices) in the USA and endorsement by UK National Societies for service provision to reduce inequity.^14 16^

This study aims to assess the impact of these changes and present an up-to-date evaluation of national MRI provision to patients with CIED in England, to assess any ongoing care gaps or inequality and to propose areas for further needed interventions to improve care.

## Methods

### Survey distribution and data collection

All National Health Service (NHS) hospitals in England with MRI were invited to participate via an online data collection instrument to assess MRI scan provision to cardiac device patients in the 12 months to January 2019. The survey has been previously described,^9^ but in brief, it comprised 21 closed response questions and limited free-text answers (online supplemental data table 1) assessing infrastructure (including the presence of onsite cardiac services), CIED scanning characteristics (MR-conditional/non-MR conditional; pacemaker/ICD), complications and volumes (categorical 0, 1–5, 6–10, 11–20, 21–50, 51–100, 101–200 and 200+ or absolute numbers). Perceived barriers to service provision/expansion and local safety protocols employed were also collected. Complications were classified into major (death, new arrhythmia, generator/lead revision) or minor (sensing changes or power-on-reset not requiring intervention beyond device reprogramming). Results were then compared with data from the previously published survey related to scans performed between January 2014 and January 2015 across the same NHS Hospital Trusts.^9^

10.1136/openhrt-2021-001598.supp1 Supplementary data

**Table 1:** Reported complications during MRI scanning in CIED patient groups (all-types) by year; 2014/15 vs 2018/19

MRI scanning complications in patients with CIED (all-types)	n (%) 2014/15	n (%) 2018/19
None	1083 (99.4%)	4891 (99.9%)
Minor	5 (0.5%)	2 (0.04%)
Major	1 (0.1%)	3 (0.1%)
Not specified	1 (0.1%)	0 (0%)

Details of complication classification are listed in online supplemental data table 2. Reported complication rates during MRI scanning for MR-conditional and non-MR conditional devices.

CIED, cardiac implantable electronic device.

Separately, to establish whether regional differences in provision of CIED scanning reflects geographical variations in clinical need, CIED implantation rates by hospital Trust and region were obtained centrally from the National Cardiac Rhythm Device Management Audit 2016–2017 (part of the National Cardiac Audit Programme to which all NHS hospitals are required to submit complete data).^5^ Regional populations were obtained from the UK Office for National Statistics,^17^ with regions defined by NHS STPs (via the NHS Shared Planning Guidance database).^18^ Ethical approval for the study was provided by the University College London Research Ethics Committee (Project ID 14287/001).

### Statistics

Categorical data are presented as absolute numbers (n) and frequencies (%). Group comparisons were performed using the χ^2^ test on GraphPad Prism V.8 (GraphPad Software, California, USA). P values of <0·05 were considered significant.

## Results

Responses were received from 212 of 223 (95%) hospitals, representing 141 of 145 (97%) of acute hospital NHS Trusts within England. Of these, 35 provided data across two of more hospital sites within a single Trust, thus, results are based on a total of 177 responses. The survey was completed by superintendent radiographers (83%), senior radiographers (10%), cardiologists (5%) and radiologists (2%).

### 2018/2019 provision of MRI for patients with CIED

#### MR conditional CIEDs

Of 112 (53%) hospitals provided MRI scans to patients with MR conditional pacemakers and 90 (43%) departments to patients with MR conditional implantable cardioverter defibrillators (ICDs).

#### Non-MR conditional CIEDs

Of 22 (10%) hospitals provided MRI to patients with non-MR conditional CIEDs, with the majority having performed fewer than five such scans. 85% of all survey responders were, however, aware of the existence of published safety protocols.

### Annual CIED MRI volume 2018–2019 and comparison with 2014–2015 data

**Figure 1:**
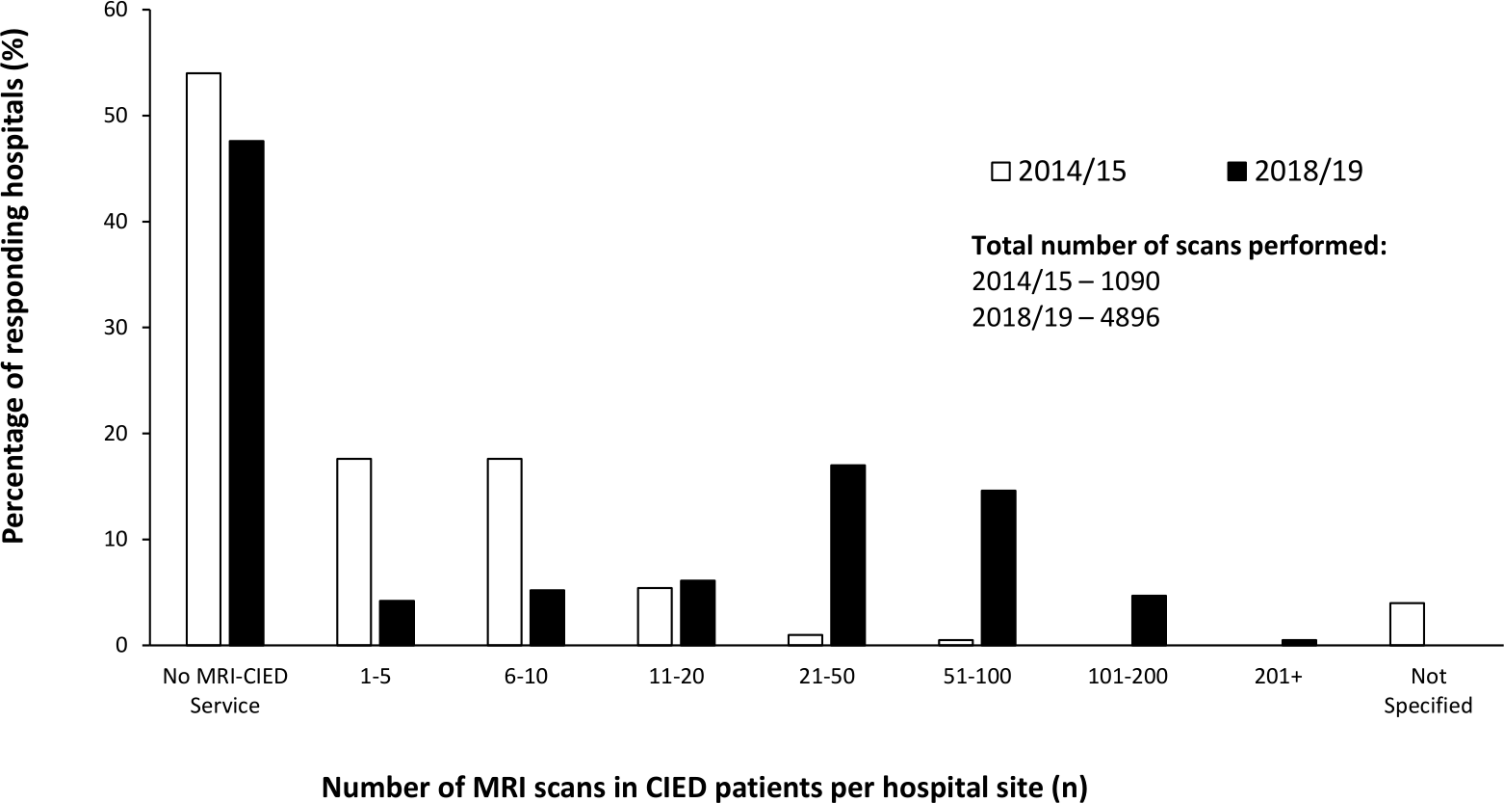
Annual MRI scan volume in patients with CIED by hospital site—comparison of 2014/15 and 2018/19 data. CIED, cardiac implantable electronic device.

There were 4896 MRI scans performed in patients with CIEDs in the year to January 2019; of which 4612 (94%) were in patients with MR conditional and 284 (5·8%) non-MR conditional devices. More than half of national provision for nonconditional device scanning was provided by just three hospitals located in London, n=154 (54% of total) scans. Of hospitals performing MRI scans in patients with CIED, median annual scan volume per hospital was 21–50 scans: range 1–210 (figure 1).

The number of NHS hospitals providing CIED–MRI services increased from 47% to 53% from 2014/2015 to 2018/2019, with non-MR conditional provision increasing from 4% to 10%. Overall scan volumes increased 4·5-fold (1090 to 4896 scans annually).

### Regional variation in supply of MRI to patients with CIED patients and relationship with estimated scan demand

**Figure 2:**
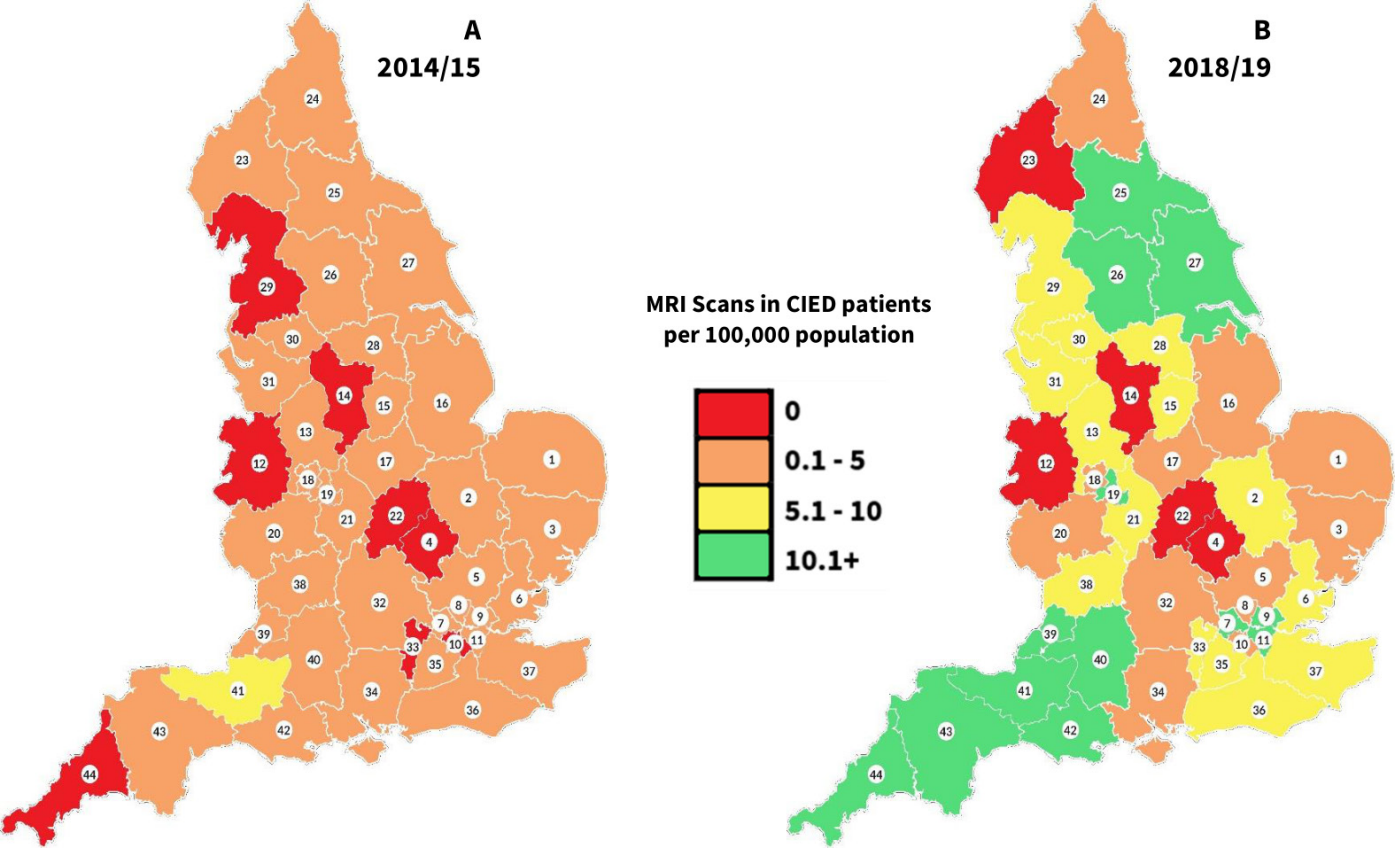
Geographical distribution of MRI scans performed in patients with CIED by STP in 2014/15 (left) and 2018/19 (right). Annual volume of MRI scans to patients with CIED performed in England demonstrating widespread geographical variation despite recent localised improvements. Total volume of CIED MRI scans performed per 12 months by STP in England by population (2014/15 data taken from Sabzevari *et al*^9^). CIED, cardiac implantable electronic device; STP, sustainabilityand transformation partnership.

There was significant regional variation in provision (figure 2). Five STPs (total population >3·5 million people and an estimated 18 750–29 000 patients with CIEDs) possessed no local provision at any NHS site within their borders.

**Figure 3:**
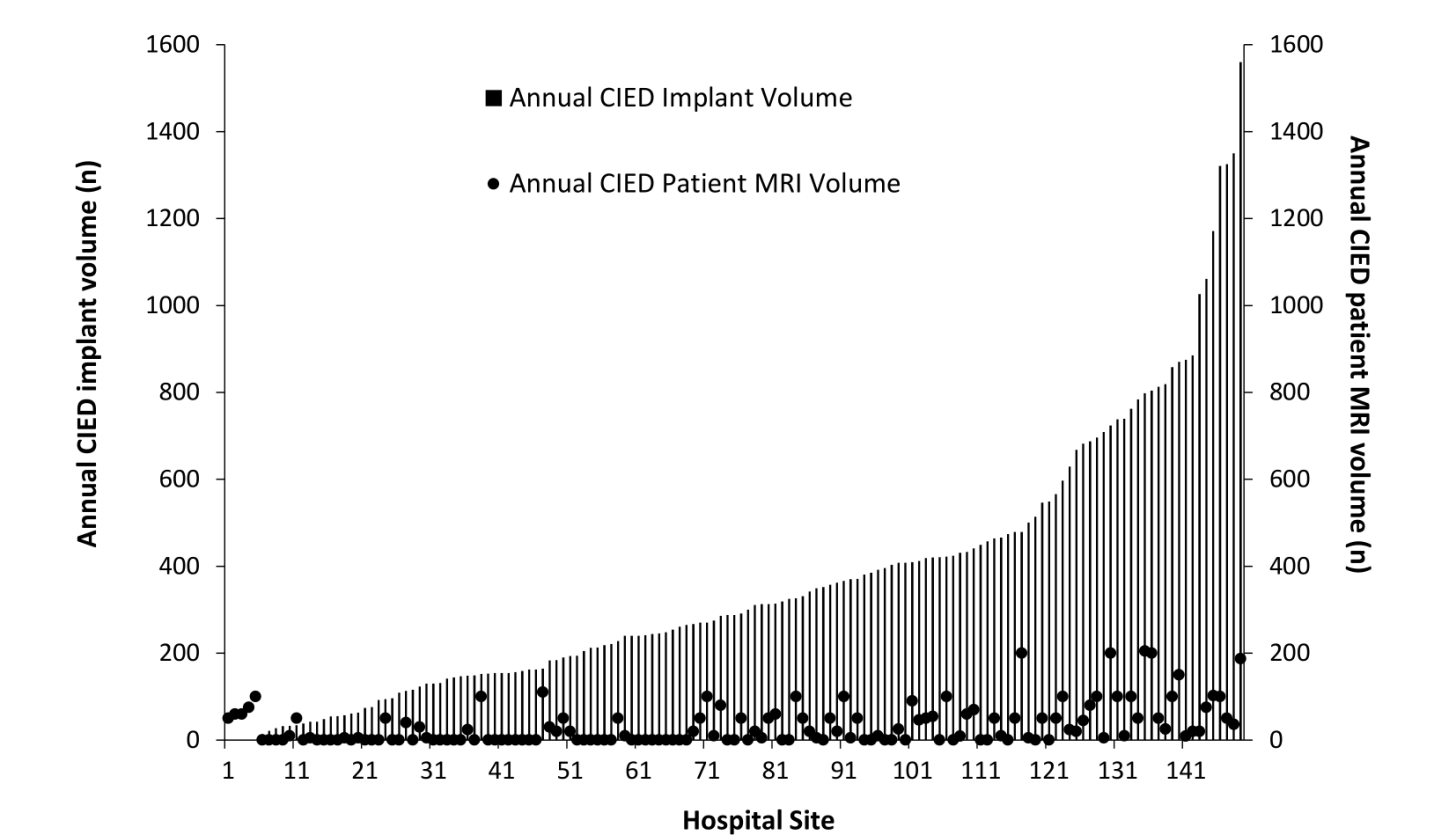
Annual CIED implant volume (bars) and CIED MRI scan volume (circles) by individual NHS hospital 2018/19. There is no relationship between the number of CIEDs implanted (demand) and the volume of MRI scans performed (supply) in patients with CIED between individual NHS Hospital Trusts. CIED, cardiac implantable electronic device; NHS, National Health Service.

Although rates of CIED device implantation vary widely between hospitals (median 288, 95% CI 240 to 336, implants per year, range 0–1560), there was no correlation between sites implanting large volumes of CIEDs and provision of MRI scans to patients with CIED (figure 3). Additionally, 64 implanting hospitals (43% sites) offered no MRI scanning for patients with CIED, despite collectively implanting in excess of 13 600 CIEDs per year (~25% of all UK CIED implants).

### MRI logistics

Most hospitals offering MRI to patients with CIEDs had cardiology or dedicated pacemaker clinics on site with 12 sites (nine of which scanned MR conditional ICDs) using visiting cardiology services from networked hospitals. All sites performing MRI in patients with non-MR conditional devices had an in-house cardiology department with pacing clinic.

### Safety data

Reported complications were very infrequent for both MR conditional and non-MR conditional devices (table 1). There were three reported major complications (complication rate 0·06%), all of which occurred in patients with MR conditional devices. One patient had ventricular fibrillation due to R on T during device interrogation outside of the scanner prior to scanning. The patient had known ischaemic cardiomyopathy and a cardiac resynchronisation therapy-pacemaker device. The patient was successfully resuscitated and underwent CIED upgrade to defibrillator. There was one reported lead displacement during scanning requiring repositioning, and at one site, the pacemaker programmer was inadvertently taken into the MRI scanner by the pacing physiologist, necessitating scanner quench with no harm to the patient or staff.

For patients with non-MR conditional CIEDs, there were no major complications and only two minor changes to right ventricular (RV) lead parameters—one change in RV lead pacing polarity; one increase in RV impedance following MRI—neither of which required further invasive intervention.

### Reported barriers to provision of MRI to patients with CIED

Of the 100 hospitals not currently offering current services, 19 planned to start scanning in the next 12 months. Twenty-three sites reported a formal arrangement with another hospital to provide this service, of which 15 stated that they had no plans to start MRI services for patients with CIED in the next 12 months. The most common stated reasons being lack of support from cardiology or pacing clinics (64%), lack of available monitoring equipment (51%), lack of training (45%), lack of scanner capacity (36%) and concerns regarding patient risk (35%).

A total of 190 (90%) hospitals do not offer MRI for patients with non-MR conditional devices. 98 (52%) reported that this might change in the future—even in hospitals not currently scanning patients with MR conditional CIEDs. Cited factors that would encourage local service delivery were multifactorial, including national multidisciplinary guidelines with representation from radiology, radiographers and medical physics (73% of respondents), greater availability to formal training (65%) and better local radiology–cardiology collaboration (64%).

## Discussion

Despite progress, accessing MRI remains challenging for patients with CIED in England, with almost half of hospitals not providing scans to patients with MRI conditional devices. Four years ago, we demonstrated an estimated 50-fold under provision of MRI scans to this patient group.^5^ Since then, changes to guidelines, pressure from national societies and increased awareness have been aimed at reducing inequity in provision.^8 11 13^ These data show some improvements with an increase in scanning volume by 4·5-fold. However, the number of centres providing cardiac device MRI services has not increased significantly over the studied period. This suggests that many hospitals are relying on the existing infrastructure currently available in other hospitals, rather than initiating their own services, evidenced by survey data showing that 23 hospitals currently have formal relationships with other hospitals to provide cardiac device MRI to their patients. It is estimated that England needs 50 000 such scans a year meaning there is still an estimated 10-fold under provision.^4 15 16^ This care gap is uneven geographically (five STPs with no provision) and greatest for patients with legacy non-MR conditional devices where three sites provide half of the national scan volume. There appears to be no relationship between supply and demand of CIED MRI scans—many sites that implant high numbers of devices still do not provide MRI scans to these patients or do so only at small volume. We found that of those centres that do not scan patients with CIED but are high-volume implanting sites (>457 implants per year, top quartile of all UK implanting centres), 25% reported lack of support from cardiology as the main barrier to providing MRI to patients with CIED. The British Heart Rhythm Society Standards for Implanting Centres^19^ have highlighted the requirement for implanting centres to provide pacing support for local MRI scanning for their patients, and, hence, hopefully should help to redress this current gap. These findings are in line with previous studies from other countries where delivery of MRI to patients with CIED is highly variable between individual centres, and patients still report challenges with accessing scans at every level from referral bias to departmental barriers.^20 21^

MRI is a fundamental component of many diagnostic and treatment pathways, and these data highlight the work that still must be done to eliminate the inequity still facing cardiac device patients. These data demonstrate that only 0·14% of the total volume of MRI scans acquired annually in England are performed in patients with CIEDS in England currently,^22^ despite their representing almost 1% of the population and a group with significant comorbidities and, hence, high clinical need for diagnostic imaging. Previous data have shown that the diagnostic yield from MRI in patients with CIED is high,^11 23^ resulting in changes to diagnosis, prognosis or clinical management in the majority of patients. The current survey also highlights that scanning is safe with very low complication rates even across patients with non-MR conditional CIED (0·06%). These data add to a growing body evidence supporting the safety of expansion of MRI services to all patients with CIED, provided strict protocols are followed.

There is significant regional and institutional variability in service provision, with patients with CIED in several areas having no access to MRI locally. Given this heterogeneity in CIED–MRI activity between institutions, it is important to understand the barriers to service development and expansion while recognising successes. Safety concerns appear no longer to be a major barrier, likely reflecting the increased body of safety data alongside more published guideline recommendations.^17^ The principal remaining barrier now relates to the logistical burden and coordinating cardiology and radiology teams. Currently, in most MRI departments, imaging is performed as per standardised protocols that obviate the need for the physical presence of a radiologist. This, combined with concerns regarding potential reduced scanner throughput with downstream impact on departmental productivity, means that many radiologists and managers fail to support initiating CIED–MRI services. Digital referral platforms that can centralise patient information and multidisciplinary decision-making may streamline the process. Hospitals scanning high volumes of device patients are generally those with large cardiology departments where collaboration is strong between the departments and where a ‘one-stop’ model can be employed to enable dedicated device MRI lists with cardiac physiologists present in the MRI department.^17^ However, these data also highlight that it is not essential to have cardiac pacing facilities on site—several hospitals are able to operate device MRI services using visiting cardiac physiologists from neighbouring hospitals/sites to perform device interrogation and reprogramming. Conversely many hospitals that implant large volumes of pacemakers and defibrillators still fail to offer MRI scans, despite clear UK guidelines requiring implanting cardiologists to support radiology departments to deliver MRI services to their patients.^14^

There is high-level consensus from leading cardiology and radiology groups that cardiac device patients must not be denied their right to equitable access to MRI. A multi-faceted approach will be required to achieve this aim and a Joint Societal Working Group for Cardiac Device MRI has been formed in the UK with representation from the National Societies of all stakeholders involved (patients, radiology, cardiology, medical physics, radiographers, referrers, NHS England). Repeated top-down recommendations to promote change have also provided impetus and mandates for change as demonstrated by the Royal College of Radiologists and British Cardiovascular Society in 2018.^16^

Other nationwide initiatives to encourage service provision include ongoing work regarding financial remuneration for scans to reflect increased scan complexity, changes to electronic requesting for scans, platforms to facilitate data transfer relating to implanted device details (including mrimypacemaker.com) and formal training of both clinicians and referrers.

## Conclusion

Almost half of the hospitals in England do not provide MRI scans to patients with MR conditional CIEDs despite awareness of technological advances and safety data. There remains an estimated 10-fold under provision despite an almost fourfold increase in scan volume over 4 years, with significant regional and institutional variation and little relationship between supply and demand. Barriers to change remain largely logistical and financial; however, these must be addressed to alleviate the current inequity in clinical care that cardiac device patients face.

## Data Availability

Data are available upon reasonable request. Metadata and hospital site-anonymised data from the survey is available following reasonable written request to the corresponding author.
