# Supplementary material for: Access to MRI for patients with cardiac pacemakers and implantable cardioverter defibrillators
Source: Open Heart. 2021 May 14;8(1):e001598. doi: 10.1136/openhrt-2021-001598 (PMC8149430; doi:10.1136/openhrt-2021-001598)
Supplement: Supplementary data [file openhrt-2021-001598supp001.pdf]

Number	Question
1	What is your role within the department
2	Which hospital is your MRI unit based in, and as part of which NHS trust
3	Does your hospital have a cardiology department, including pacing clinic, on site
4	Is your unit aware that non-MR conditional devices can be scanned using appropriate safety measures
5	Does your unit scan patients with; **MR-conditional pacemakers** (Y/N), **MR-conditional defibrillators** (Y/N), **non-MR conditional devices** (Y/N)
6	Where do you scan device patients
7	Where is device re-programming performed
8	Does your unit scan the thorax in cardiac device patients
9	How many scans has your unit performed in patients with in-situ pacemakers/defibrillators (of all types) in the preceding 12 months
10	How many of your scans are in patients with non-MRI conditional pacemakers/defibrillator systems
11	How many scans do you anticipate delivering in the upcoming 12 months
12	*Written standard operating procedure in place* | *CXR prior to MRI to look for abandoned leads* | *Request discussed with cardiologist/ pacing clinic prior to booking* | *ALS trained personnel on site*<br>*Cardiologist present on site* | *Cardiologist present in department* | *Cardiac device physiologist/ technician present on site* | *Cardiac device physiologist/ technician present in department*<br>*Radiologist present in department* | *Continuous ECG monitoring* | *Continuous pulse oximetry monitoring* | *Other (please specify)*<br>Which of the following precautions are taken when scanning patients with MRI conditional devices? Tick as many as appropriate.
13	*Written confirmation that no other imaging modality will answer clinical question* | *Written patient consent* | *Discussion with cardiologist regarding individual risk of MRI* | *MDT discussion*<br>*Written confirmation that scan results will change management* | *Cardiologist present in the department* | *Local list of indications which are always appropriate (e.g. suspected cord compression)* | *Other (please specify)*<br>What additional precautions are taken when scanning patients with non-MRI conditional devices? Tick as many as appropriate.
14	Have you scanned any of the following patients; abandoned/capped leads, devices implanted <6 weeks, abdominal pacing systems, epicardial pacing leads, non-MRI-conditional pacemaker dependent ICD patients?
15	Have you ever had any complications scanning any pacemaker/defibrillator patients? Please see Supplementary Data for potential complication details.
16	Did these complications arise whilst adhering to local protocols?
17	What is the reason(s) for not scanning patients with MR-conditional devices? Tick as many as appropriate.<br>- Unaware of MR-conditional devices<br>- Concerns regarding patient risk<br>- Lack of training<br>- Lack of support from cardiology/pacing clinic<br>- Lack of available monitoring equipment<br>- Lack of tariff/concerns regarding funding<br>- Lack of capacity on scanner<br>- MRI provided by 3rd party who do not permit device scanning<br>- Device MRI provided by another hospital within the Trust<br>- Lack of time to review patient/device data before scan<br>- Scan unimportant/no referrals
18	Would you ever feel comfortable scanning non-MR-conditional devices
19	What would encourage you/your unit to start scanning these patients; formal training, national guidelines (e.g. Royal College of Radiology, British Cardiovascular Society), better cardiology-radiology collaboration, better support from pacing technicians, dedicated tariff/funding agreement, logistical support to review device information, more referrals.
20	Would you be interested in further training on MRI scanning in patients with cardiac devices

Supplementary Data Table 1

Online questionnaire sent to all NHS MRI units in England.

Category	Complication

**Major**	- Death<br>- Generator failure requiring replacement<br>- Significant battery depletion requiring generator replacement<br>- Lead displacement requiring repositioning<br>- Inhibition of necessary pacing<br>- Sustained arrhythmia attributable to current induction in pacemaker or ICD system leads (i.e. co-incident with MRI sequence initiation, rhythmic pulse sequences, or sequence termination)<br>- Inappropriate delivery of anti-tachycardia pacing or shocks
**Minor**	- Power-on-reset<br>- Significant lead parameter (pacing threshold, impedance or sensing) changes requiring changes in programmed sensitivity or pacing output

Supplementary Data Table 2

Classification of potential complications from MRI scanning in CIED patients (derived from the MagnaSafe Registry (19)).

			Total MRI Scan Number in CIED Patients		Total MRI Scan Number in CIED Patients	
**Map Number**	**Sustainability & Transformation Partnership**	**STP Population (2016)**	**2014/15**	**2018/19**	**2014/15**	**2018/19**
**1**	Norfolk and Waveney	1,100,000	10	20	0.91	1.82
**2**	Cambridgeshire and Peterborough	900,000	40	86	4.44	9.56
**3**	Suffolk and North East Essex	1,000,000	20	20	2.00	2.00
**4**	Milton Keynes, Bedfordshire and Luton	1,000,000	0	0	0.00	0.00
**5**	Hertfordshire and West Essex	1,600,000	30	70	1.88	4.38
**6**	Mid and South Essex	1,200,000	20	75	1.67	6.25
**7**	North West London	2,500,000	80	429	3.20	17.16
**8**	North London	1,600,000	20	70	1.25	4.38
**9**	North East London	2,200,000	40	362	1.82	16.45
**10**	South West London	1,700,000	0	15	0.00	0.88
**11**	South East London	2,000,000	20	250	1.00	12.50
**12**	Shropshire, Telford and Wrekin	500,000	0	0	0.00	0.00
**13**	Staffordshire & Stoke-on-Trent	1,200,000	20	102	1.67	8.50
**14**	Derbyshire	1,000,000	0	0	0.00	0.00
**15**	Nottingham & Nottinghamshire	1,100,000	10	100	0.91	9.09
**16**	Lincolnshire	800,000	20	20	2.50	2.50
**17**	Leicester, Leicestershire and Rutland	1,100,000	50	50	4.55	4.55
**18**	The Black Country & West Birmingham	1,500,000	50	20	3.33	1.33
**19**	Birmingham & Solihull	1,300,000	20	200	1.54	15.38
**20**	Herefordshire and Worcestershire	800,000	20	10	2.50	1.25
**21**	Coventry and Warwickshire	1,000,000	20	90	2.00	9.00
**22**	Northamptonshire	800,000	0	0	0.00	0.00
**23**	West, North and East Cumbria	300,000	10	0	3.33	0.00
**24**	Northumberland, Tyne and Wear, and North Durham	1,700,000	10	75	0.59	4.41
**25**	Durham, Darlington, Teesside, Hambleton, Richmond and Whitby	1,200,000	20	200	1.67	16.67
**26**	West Yorkshire and Harrogate	2,500,000	60	255	2.40	10.20
**27**	Humber, Coast and Vale	1,700,000	30	275	1.76	16.18
**28**	South Yorkshire and Bassetlaw	1,500,000	10	100	0.67	6.67
**29**	Lancashire and South Cumbria	1,700,000	0	124	0.00	7.29
**30**	Greater Manchester	2,800,000	100	250	3.57	8.93
**31**	Cheshire and Merseyside	2,600,000	70	260	2.69	10.00
**32**	Buckinghamshire, Oxfordshire and Berkshire West	1,800,000	20	85	1.11	4.72
**33**	Frimley Health	800,000	0	46	0.00	5.75
**34**	Hampshire and the Isle of Wight	1,900,000	20	79	1.05	4.16
**35**	Surrey Heartlands	900,000	10	50	1.11	5.56
**36**	Sussex and East Surrey	1,800,000	30	140	1.67	7.78
**37**	Kent and Medway	1,900,000	30	160	1.58	8.42
**38**	Gloucestershire	600,000	10	50	1.67	8.33
**39**	Bristol, North Somerset and South Gloucestershire	1,000,000	50	120	5.00	12.00
**40**	Bath and North East Somerset, Swindon and Wiltshire	900,000	10	104	1.11	11.56
**41**	Somerset	600,000	40	80	6.67	13.33
**42**	Dorset	800,000	20	170	2.50	21.25

**43**	Devon	1,200,000	20	184	1.67	15.33
**44**	Cornwall and the Isles of Scilly	600,000	0	100	0.00	16.67

Supplementary Data Table 3

National provision of MRI scans to CIED patients by sustainability and transformation partnership. This supplementary data table demonstrates the raw data used for Figure 3.
